# Exploring knowledge, attitudes, and practices towards artificial intelligence among health professions’ students in Jordan

**DOI:** 10.1186/s12911-023-02403-0

**Published:** 2023-12-14

**Authors:** Walid Al-Qerem, Judith Eberhardt, Anan Jarab, Abdel Qader Al Bawab, Alaa Hammad, Fawaz Alasmari, Badi’ah Alazab, Daoud Abu Husein, Jumana Alazab, Saed Al-Beool

**Affiliations:** 1 Department of Pharmacy, Faculty of Pharmacy, Al-Zaytoonah University of Jordan, 11733 Amman, Jordan; 2 School of Social Sciences, Humanities and Law, Department of Psychology, Teesside University, TS1 3BX Middlesbrough, UK; 3 College of Pharmacy, Al Ain University, 64141 Abu Dhabi, UAE; 4 AAU Health and Biomedical Research Center, Al Ain University, 112612 Abu Dhabi, United Arab Emirates; 5 Department of Clinical Pharmacy, Faculty of Pharmacy, Jordan University of Science and Technology, 22110 Irbid, Jordan; 6 Department of Pharmacology and Toxicology, College of Pharmacy, King Saud University, 12372 Riyadh, Saudi Arabia; 7 School of Medicine, The University of Jordan, 11910 Amman, Jordan

**Keywords:** Artificial intelligence, Medicine, Students, Jordan, Education, Pharmacy

## Abstract

**Introduction:**

The integration of Artificial Intelligence (AI) in medical education and practice is a significant development. This study examined the Knowledge, Attitudes, and Practices (KAP) of health professions’ students in Jordan concerning AI, providing insights into their preparedness and perceptions.

**Methods:**

An online questionnaire was distributed to 483 Jordanian health professions’ students via social media. Demographic data, AI-related KAP, and barriers were collected. Quantile regression models analyzed associations between variables and KAP scores.

**Results:**

Moderate AI knowledge was observed among participants, with specific understanding of data requirements and barriers. Attitudes varied, combining skepticism about AI replacing human teachers with recognition of its value. While AI tools were used for specific tasks, broader integration in medical education and practice was limited. Barriers included lack of knowledge, access, time constraints, and curriculum gaps.

**Conclusions:**

This study highlights the need to enhance medical education with AI topics and address barriers. Students need to be better prepared for AI integration, in order to enable medical education to harness AI’s potential for improved patient care and training.

**Supplementary Information:**

The online version contains supplementary material available at 10.1186/s12911-023-02403-0.

## Introduction

Artificial Intelligence (AI) is a comprehensive term encompassing the technology that enables computer hardware and software to mimic intelligent human behavior and reach human-level performance [1]. AI-powered technologies have been implemented in many professions such as law, finance, computer science, and industrial manufacturing [2]. This rapid expansion and evolution of AI has allowed it to extend to the medical field. For instance, AI has been integrated into oncology for cancer diagnosis and grading [3], as well as into endoscopes in gastroenterology to detect and diagnose pathological lesions [4]. Moreover, AI has found its way into the education and training of health professions’ students. To further illustrate, a computer-aided learning system has been constructed to help health professions’ students gain diagnostic experience by training this machine learning model using numerous clinical cases [5]. In addition, Virtual Patients have been considered an ideal virtual counseling platform for undergraduate nursing students to help develop their communication skills before engaging with real-life patients and other healthcare providers during their clinical posting [6, 7].

It is apparent that AI will be highly integrated into the medical field, but its future impact on health professions’ students remains ambiguous. Various studies have shown that AI could leave students of certain medical professions (e.g., radiology) less enthusiastic about the field [8]. Furthermore, resources for the implications of AI in medical training and education remain limited globally, particularly in the Middle East region [9].

While several studies have evaluated the Knowledge, Attitudes, and Practices (KAP) of students across diverse medical disciplines toward AI, it is worth noting that such examinations are still notably absent in the context of Jordan [2, 9, 10]. This scarcity is noteworthy, especially when considering Jordan’s recognized status as a regional center for medical care and medical tourism, characterized by an annual revenue of $1.1 billion and a consistent annual growth rate of approximately 10% in the number of foreign patients [11, 12]. Recognizing the growing importance of AI in its incorporation in medical education for equipping future healthcare professionals with the skills and knowledge necessary to navigate the increasingly developing fields [6]. Therefore, the primary objective of our study is to determine KAP in relation to AI among this population in Jordan. Additionally, we explored students’ perspectives on the potential role of AI as a teaching tool and their attitudes toward implementing it as a subject in the taught curriculum. By examining the KAP of Jordanian health professions’ students, we aim to enrich the understanding of AI’s significance in a region where medical care and medical tourism play a pivotal role in the national economy.

This study provides context-specific insights and implications, filling an existing gap in the literature while adding depth to the international discourse on AI in healthcare education.

## Methods

An online questionnaire in the English language, as it is the only teaching language in medical field’s colleges, was constructed using Google Forms, and distributed to students across different faculties within the health professions (medicine/dentistry, pharmacy/pharmD, and others which included nursing and physical therapy) in the period from June 2023 through August 2023 through Jordanian social media groups on Facebook dedicated to health professions’ students. The introductory information on the questionnaire emphasized that participation was voluntary and that the collected data would be kept confidential and to be used only for research purposes. Ethical approval was obtained from the Institutional Review Board and the Deanship of Research at Al-Zaytoonah University of Jordan.

### Data collection and study instruments

A thorough literature review [2, 9, 10] was conducted as part of constructing the questionnaire (Supplementary material: S1). The questionnaire consisted of five sections. The first section gathered participants’ demographic information, including age, gender, type of university (public or private), type of college, and academic year of study. The subsequent sections evaluated participants’ AI KAP, while the final section explored potential barriers that might prevent students from using AI in their daily activities. The knowledge-assessing part consisted of 7 items, the attitude-assessing part of 10 items, the practice-assessing part of 7 items, and the part examining barriers towards using AI of 8 items. The total knowledge score was calculated by awarding 1 point for each affirmative response (Yes) and 0 points for negative responses (No), yielding a total possible score of 7. The scoring for both the attitude and the practice domains used a 5-point Likert scale ranging from ‘strongly disagree’ (1 point) to ‘strongly agree’ (5 points) for attitudes, and from ‘never’ (1 point) to ‘all the time’ (5 points) for practices, with a total possible score of 50 and 35, respectively. The barriers section consisted of 7 potential factors that could prevent students from integrating AI into their daily life, along with a “none” choice in case no barriers were recognized by the participant. These barriers included not knowing enough about AI, having insufficient access to AI or not the right technical equipment, concerns about ethics and privacy, not having enough time due to educational commitments, AI being perceived as too complicated, not learning about AI as part of the educational curricula, and not having places or opportunities to learn and practice AI skills.

### Instrument validation

An expert panel that included four professors in medicine, dentistry, pharmacy, and nursing confirmed the content validity of the questionnaire. A pilot study with 30 students was conducted to ensure that all questions in the instrument were clear; their data was not included in the final analysis. The internal consistency of the three composed latent variables (knowledge, attitude, and practice) were confirmed by computing Cronbach’s alphas.

### Sample size calculations and data collection method

Convenience sampling was used in the current study [13]. Morgan equation was applied with a 95% significance level and a 5% margin of error to calculate the minimum required sample size (385 participants). The current study included 483 students.

### Statistical analysis

SPSS version 28.0 was used to analyze the data. Frequencies and percentages were used to present all categorical variables, and median (95% CI) for continuous variables. The distribution of the data was evaluated by examining histograms and Q-Q plots which indicated that the data was not normally distributed; therefore, nonparametric tests were conducted. To determine variables associated with KAP scores, three quantile regression models were constructed. For the knowledge-score model the independent variables included age, gender, university type (private vs. public) and college type. The attitude-score model included all the previously mentioned variables in addition to the knowledge score as independent variables, while the practice-score model also included knowledge and attitude scores as independent variables. The significance level was determined at *p* < 0.05.

## Results

The present study enrolled 483 students (63.8% female and 36.2% male), with a median age of 21 (range 21–22). Almost half of the participants were studying medicine/dentistry (47.6%) and 41.6% were studying for a bachelor of pharmacy (PharmB) or a doctor of pharmacy (PharmD), while 10.8% were studying in other medical fields. More than two thirds of participants were from public universities (67.9%). See Table 1 for demographic characteristics of the sample.

**Table 1:** Participants’ sociodemographic characteristics

		Median (95% Cl) or Frequency (%)
Age (in years)		21 (21–22)
Gender	Female	308 (63.8%)
	Male	175 (36.2%)
Type of university	Public	328 (67.9%)
	Private	155 (32.1%)
Type of college	Medicine/Dentistry	230 (47.6%)
	PharmB/PharmD	201 (41.6%)
	Other	52 (10.8%)
Academic year	1st year	87 (18%)
	2nd year	91 (18.8%)
	3rd year	72 (14.9%)
	4th year	89 (18.4%)
	5th year	129 (26.7%)
	6th year	15 (3.1%)

Participants’ responses to the knowledge items are presented in Table 2. The item most frequently answered with ‘yes’ was “AI requires a lot of labeled data to learn (data already processed by a human)” (66.8%) followed by “I understand the barriers of applying AI in medicine” (58.9%) and “Do you know any application of AI in your field of interest?” (58.9%). On the other hand, the items least frequently answered with ’yes’ were “Have you ever been taught about AI in your undergraduate studies?” and “Have you attended any previous online/offline courses regarding AI?” (25.5% and 30.3% respectively). The median knowledge score was 3 (range 3–4) out of a maximum possible score of 7. Cronbach’s alpha (0.82) indicated acceptable internal consistency.

**Table 2:** Frequencies of participants’ responses to knowledge items

	No (%)	Yes (%)
Do you have a solid knowledge of the basics of AI?	242 (50.5%)	237 (49.5%)
Do you know what deep learning/machine learning is?	300 (62.6%)	179 (37.4%)
Do you know any application of AI in your field of interest?	197 (41.1%)	282 (58.9%)
Have you attended any previous online/offline courses regarding AI?	334 (69.7%)	145 (30.3%)
Have you ever been taught about AI in your undergraduate studies?	357 (74.5%)	122 (25.5%)
AI requires a lot of labeled data to learn (data already processed by a human).	159 (33.2%)	320 (66.8%)
I understand the barriers of applying AI in medicine.	197 (41.1%)	282 (58.9%)

Table 3 presents participants’ responses to the attitude items. The most frequent disagree/strongly disagree responses were provided for the items “I believe human teachers will be replaced in the foreseeable future” (34.1%) followed by “Clinical AI will be more accurate than physicians” (28.2%), while the least frequent disagree/strongly disagree responses were provided for items “I believe healthcare students should learn the basics of AI” and “I believe ethical implications of AI must be understood by different students” (4.5% and 4.8% respectively). The median for the attitude score was 37 (range 37–38) out of a maximum possible score of 50. The Cronbach’s alpha (0.87) indicated acceptable internal consistency.

**Table 3:** Frequencies of participants’ responses to attitude items

	Strongly Disagree (%)	Disagree (%)	Neutral (%)	Agree (%)	Strongly Agree (%)
I believe healthcare students should learn the basics of AI	5 (1%)	17 (3.5%)	98 (20.5%)	202 (42.2%)	157 (32.8%)
I believe AI will be a highly required tool in my field	6 (1.3%)	22 (4.6%)	118 (24.6%)	182 (38%)	151 (31.5%)
I believe ethical implications of AI must be understood by different students	4 (0.8%)	19 (4%)	99 (20.7%)	189 (39.5%)	168 (35.1%)
I believe AI will revolutionize the educational system	5 (1%)	24 (5%)	128 (26.7%)	146 (30.5%)	176 (36.7%)
I believe human teachers will be replaced in the foreseeable future	53 (11.1%)	110 (23%)	179 (37.4%)	90 (18.8%)	47 (9.8%)
I believe the upcoming developments in the educational system will excite me	12 (2.5%)	28 (5.8%)	151 (31.5%)	180 (37.6%)	108 (22.5%)
I believe AI should be a part of the training system among students of medical fields	5 (1%)	30 (6.3%)	133 (27.8%)	196 (40.9%)	115 (24%)
Clinical AI will be more accurate than physicians	41 (8.6%)	94 (19.6%)	196 (40.9%)	97 (20.3%)	51 (10.6%)
I believe some specialties are more prone to be replaced by AI than others	16 (3.3%)	50 (10.4%)	165 (34.4%)	153 (31.9%)	95 (19.8%)
I believe AI would increase the percentage of errors in diagnosis	15 (3.1%)	76 (15.9%)	206 (43%)	123 (25.7%)	59 (12.3%)

The most frequently reported practices (answered with “all the time/most of the time”) were using AI for spelling and grammar checking and using AI to conduct research (38.2% and 35.5%), while the least-reported practices were using AI for personal choices/career guidance (26.5%) followed by using AI to prepare for exams (29.3%). The median for the practice items was 21 (range 21–23) out of a maximum possible score of 35. Cronbach’s alpha of 0.95 indicated good internal consistency (Table 4).

**Table 4:** Frequencies of participants’ responses to practice items

	Never (%)	Rarely (%)	Often (%)	Most of the time (%)	All the time (%)
How frequently do you use AI to prepare for your exams?	114 (23.8%)	103 (21.5%)	122 (25.5%)	100 (20.9%)	40 (8.4%)
How frequently do you use AI to prepare for your homework/assignments?	81 (16.9%)	98 (20.5%)	134 (28%)	115 (24%)	51 (10.6%)
How frequently do you use AI to conduct your research?	92 (19.2%)	80 (16.7%)	137 (28.6%)	118 (24.6%)	52 (10.9%)
How frequently do you use AI for idea generation and brainstorming?	108 (22.5%)	80 (16.7%)	129 (26.9%)	104 (21.7%)	58 (12.1%)
How frequently do you use AI for personal choices/career guidance?	137 (28.6%)	105 (21.9%)	110 (23%)	88 (18.4%)	39 (8.1%)
How frequently do you use AI for spelling and grammar checking?	93 (19.4%)	71 (14.8%)	132 (27.6%)	107 (22.3%)	76 (15.9%)
How frequently do you use AI for personality development and other skills?	122 (25.5%)	89 (18.6%)	121 (25.3%)	91 (19%)	56 (11.7%)

Table 5 displays barriers hindering the use of AI, the results show that the most-reported reasons were lack of knowledge and expertise, followed by lack of time due to educational burden, and lack of access/technical equipment (52.8%, 43.1%, and 42.4% respectively). On the other hand, ethical and privacy concerns were the least frequently reported barrier (34.2%).

**Table 5:** Barriers hindering the use of AI

	Frequency (%)
Lack of knowledge and expertise	255 (52.8%)
Lack of access/technical equipment	205 (42.4%)
Ethical and privacy concerns	165 (34.2%)
Lack of time due to educational burden	208 (43.1%)
Complexity of AI	184 (38.1%)
Limited integration in educational curricula	177 (36.7%)
Lack of teaching centers and hands-on applications	181 (37.5%)

Three quantile regression models were built to assess variables associated with knowledge, attitude, and practice scores. The results revealed that type of college was significantly associated with the knowledge and the attitude scores as participants who were studying medicine/dentistry had lower knowledge and attitude scores than those who were studying PharmB/PharmD (Coefficient = -0.994, 95% Cl (-1.706, -0.282), *p* = 0.006 and coefficient = -1.573, 95% Cl (-3.094,-0.052), *p* = 0.043 respectively) and those who were studying other medical fields had lower knowledge and attitude scores compared to those who were studying PharmB/PharmD (coefficient = -3.992, 95% Cl (-4.899 - -3.086), p < 0.001 and coefficient = -3.130, 95% Cl ( -5.110, -1.151), p = 0.002 respectively). Knowledge scores were significantly positively associated with attitude and practice scores (coefficient = 1.286, 95% Cl (1.057, 1.516), *p* < 0.001 and coefficient = 1.489, 95% Cl (1.098, 1.881), *p* < 0.001 respectively). Furthermore, attitude scores were significantly positively associated with practice scores (Coefficient = 0.423, 95% Cl (0.278, 0.567), *p* < 0.001).

## Discussion

The integration of AI into various medical fields has garnered significant attention due to its potential to enhance diagnostic accuracy, optimize treatment plans [14, 15], and revolutionize the educational landscape. This study aimed to explore the KAP of health professions’ students in Jordan toward AI, shedding light on their perceptions, expectations, and concerns regarding AI integration into medical education.

The findings from this study revealed a moderate level of knowledge and awareness among the surveyed health professions’ students regarding AI. Notably, participants exhibited a strong understanding of certain aspects of AI, such as the requirement for labeled data for AI learning and the identification of barriers to AI implementation in medicine. However, a significant proportion of students reported limited exposure to formal AI education within their curriculum. Low or moderate levels of knowledge of AI and exposure within the curriculum has also been reported in previous research in other cultural contexts [16, 17]. This underscores a potential gap between the rapid advancements in AI technology and its integration into medical education in the Jordanian context.

Attitudes of Jordanian students in the medical field toward AI were multifaceted. While a considerable number of participants expressed skepticism about the potential replacement of human teachers and the superior accuracy of clinical AI over physicians, a substantial proportion also endorsed the importance of health professions’ students acquiring foundational knowledge about AI. These mixed attitudes may stem from concerns about the implications of AI on traditional medical education and practice, such as the fear of job displacement, the impact on the doctor-patient relationship, and concerns about patient safety. Additionally, cultural and contextual factors may contribute to these attitudes. In a similar vein, a survey with health professions’ students in Turkey revealed mixed attitudes toward AI, finding that most students saw AI as an assistive technology that could facilitate physicians’ access to information, patients to healthcare, and reduce errors, but half of the participants were concerned about the possible reduction in the services of physicians, and expressed a need for an update on the medical curriculum, according to necessities in transforming healthcare driven by artificial intelligence [18]. These concerns may be rooted in the uncertainty surrounding AI’s role in healthcare and the need for clear guidance and education on the integration of AI into medical practice. Other research, conducted in Kuwait, found overall positive attitudes towards AI in medical education and a consensus that AI-based medical practice is required [19]. These differences in attitudes across regions could be influenced by variations in the readiness and exposure of these countries to AI in healthcare, as well as the cultural and educational contexts specific to each region.

These mixed attitudes and perspectives suggest a nuanced outlook on AI’s role in medical education and patient care. Interestingly, in the present study ethical considerations surrounding AI were deemed of lower concern, possibly reflecting a need for enhanced awareness and education on the ethical implications of AI application in healthcare. The limited concern for ethical aspects could be attributed to several factors. Firstly, it may stem from a lack of comprehensive understanding among the participants regarding the ethical dilemmas and challenges posed by AI in healthcare. Many health professions’ students might not have been exposed to extensive training on AI ethics in their curricula. Secondly, it could be related to the prioritization of other more immediate and tangible concerns, such as the impact on their own roles as future healthcare professionals or the technical aspects of AI integration. However, the relative neglect of ethical considerations in AI implementation should not be overlooked.

The current study illuminated that students of various medical fields in Jordan have begun to incorporate AI into their daily practices, albeit to varying extents. The most commonly reported AI applications were spell and grammar checking, as well as AI-assisted research. These applications primarily serve as tools for improving academic performance and research efficiency. However, the utilization of AI for more clinical or career-oriented purposes, such as exam preparation and personal decision-making, appeared less prevalent. Similarly, to the present study, a recent systematic review on attitudes, knowledge, and skills towards AI among healthcare students found that most studies reported limited skills in working with AI [16]. These findings collectively suggest that Jordanian health professions’ students are embracing AI tools for specific tasks, particularly related to academic and research-related activities. Yet there remains untapped potential for AI integration in broader aspects of their medical education and future careers.

The present study identified several perceived barriers hindering the seamless integration of AI into the educational journey of health professions’ students in Jordan. Lack of knowledge and expertise, coupled with limited access to necessary technical equipment, emerged as primary obstacles. Moreover, concerns related to the time demands of academic studies, the perceived complexity of AI technology, and its limited integration within educational curricula were also noteworthy challenges. Previous studies have examined barriers to the acceptance and adoption of AI by healthcare professionals [20], but have not focused extensively on these barriers in health professions’ students. The barriers identified in the current study collectively underscore the need for comprehensive strategies to bridge the AI knowledge gap and facilitate the effective incorporation of AI into medical education.

The insights gained from the present study have important implications for medical education in Jordan and beyond. To harness the full potential of AI, educational institutions should consider revising curricula to incorporate AI-related topics, ensuring that health professions’ students are equipped with the necessary knowledge and skills to navigate the evolving healthcare landscape. This revision should encompass both theoretical and practical aspects of AI, including its applications in healthcare, ethical considerations, and hands-on experience with AI tools. To enhance the integration of AI within the medical field, proactive steps should be taken to incorporate AI into medical school curricula. This can be achieved through the development of dedicated AI courses or modules that address AI’s role in medicine, its practical applications, and its implications for patient care. These courses should provide students with a deeper comprehension of AI algorithms and enable them to optimize its utilization in their future medical practice. Furthermore, partnerships with AI industry experts and organizations can provide students with real-world exposure and experience, enriching their understanding of AI’s role in healthcare. This approach would empower medical professionals with a deeper comprehension of AI algorithms and enable them to optimize their utilization [21]. In addition to the technical aspects of AI education, it is imperative to address concerns related to the potential replacement of human teachers and ethical considerations. Educational institutions should prioritize promoting a balanced perspective on AI’s role in healthcare, emphasizing the complementarity of AI with human expertise rather than its substitution. Ethical discussions should be integrated into the curriculum to ensure that students are well-prepared to navigate the ethical challenges posed by AI in healthcare.

These practical steps for incorporating AI-related topics into medical education would help equip future medical professionals with the knowledge and skills needed to embrace AI as a valuable tool in their practice and adapt to the evolving healthcare landscape effectively.

### Strengths and limitations

While this study contributes valuable insights, certain limitations should be acknowledged. The use of convenience sampling may introduce selection bias, potentially limiting the generalizability of findings. However, convenience sampling is a commonly employed method in many well-structured cross sectional KAP survey [22–25]. Future research could employ diverse sampling methods to further explore the evolving perceptions of health professions’ students toward AI over time and across different cultural contexts. While the questionnaire used in the present study yielded valuable data, it may not have fully captured the multidimensional nature of health professions’ students’ AI-related knowledge and attitudes. With our study being exploratory, the findings that emerged point to a need for future research employing qualitative methods, in the form of interviews and/or focus groups, to gain deeper insights into health professions’ students’ knowledge, attitudes and practices in relation to AI.

Although the tool used in the present study underwent several validation steps which included a thorough review of the questionnaire by an expert panel to assess its face and content validity, the clarity of the questions was also verified in a pilot study that included 30 students. Furthermore, the internal consistency and reliability of the questionnaire were confirmed by computing Cronbach’s alpha, a widely recognized measure of questionnaire reliability. Nevertheless, other validation steps including test-retest reliability may have further improved the validity and reliability of the instrument.

However, several strengths of this study need to be pointed out. Firstly, the study introduces a novel perspective by addressing a critical gap in the existing literature through an investigation into the KAP of health professions’ students in Jordan regarding AI. This contribution is particularly noteworthy due to the limited research available in the Middle East region, specifically in the context of AI’s integration into medical education.

The comprehensive nature of the questionnaire, which covered demographic information, AI-related KAP, and potential barriers, allowed for a holistic understanding of the students’ viewpoints. Prior to the main data collection, a pilot study was conducted with a small group of students. This preliminary phase ensured the clarity and comprehensibility of the questionnaire items, contributing to the overall rigor of the survey design and data collection process.

## Conclusion

The present study sheds light on the KAP of health professions’ students in Jordan toward AI, offering a comprehensive understanding of their knowledge, attitudes, and practices. The study’s findings carry direct implications for medical education in Jordan, offering valuable insights into the readiness and perceptions of health professions’ students regarding AI. There is currently little consensus on what and how to teach AI in medical education [22]. The insights from the present study have the potential to guide curriculum development in Jordan, thereby better preparing future healthcare professionals to embrace AI technologies.

As AI continues to reshape the healthcare landscape, the knowledge, attitudes and practices of health professions’ students will play a pivotal role in shaping its impact on patient care and medical education.

### Electronic supplementary material

Below is the link to the electronic supplementary material.

Supplementary Material 1

## Data Availability

The datasets analyzed for this study can be found in the Zenodo (10.5281/zenodo.8229503).

## Abbreviations

KAP: Knowledge attitude and practice
AI: Artificial intelligence
PharmB: Bachelor of Pharmacy
PharmD: Doctor of Pharmacy
