# Supplementary material for: Exploring knowledge, attitudes, and practices towards artificial intelligence among health professions’ students in Jordan
Source: BMC Med Inform Decis Mak. 2023 Dec 14;23:288. doi: 10.1186/s12911-023-02403-0 (PMC10722664; doi:10.1186/s12911-023-02403-0)
Supplement: Supplementary file 1 — Supplementary Material 1 [file 12911_2023_2403_MOESM1_ESM.docx]

**Supplementary Material**

**S1:**

**Demographics**

- How old are you?
- What gender do you identify as?

Female

Male

- What is your current living situation?

Living alone

Living with others

- What classification of university do you attend?

Government/public university

Private university

- Which college are you affiliated with?

Medicine/ dentistry

Pharmacy (BPharm/PharmD)

Other medical field (Nursing/applied medical sciences)

- What is your current year of study?

1^st^ year

2^nd^ year

3^rd^ year

4^th^ year

5^th^ year

6^th^ year

**Knowledge**

|  | **Yes** | **No** |
| --- | --- | --- |
| Do you have a solid knowledge of the basics of AI? |  |  |
| Do you know what deep learning/machine learning is? |  |  |
| Do you know any application of AI in your field of interest? |  |  |
| Have you attended any previous online/offline courses regarding AI? |  |  |
| Have you ever been taught about AI in your undergraduate studies? |  |  |
| AI requires a lot labeled data to learn (data already processed by a human) |  |  |
| I understand the barriers of applying AI in medicine |  |  |

**Attitude**

|  | **Strongly agree** | **Agree** | **Neutral** | **Disagree** | **Strongly disagree** |
| --- | --- | --- | --- | --- | --- |
| I believe healthcare students should learn the basics of AI |  |  |  |  |  |
| I believe AI will be a highly required tool in my field |  |  |  |  |  |
| I believe ethical implications of AI must be understood among different students |  |  |  |  |  |
| I believe AI will revolutionize the educational system |  |  |  |  |  |
| I believe human teachers will be replaced in the foreseeable future |  |  |  |  |  |
| I believe the upcoming developments in the educational system will excite me |  |  |  |  |  |
| I believe AI should be a part of the training system among students of medical fields |  |  |  |  |  |
| Clinical AI will be more accurate than physicians |  |  |  |  |  |
| I believe some specialties are more prone to be replaced by AI than others |  |  |  |  |  |
| I believe AI would increase the percentage of errors in diagnosis |  |  |  |  |  |

**Practices**

|  | **All the time** | **Most of the time** | **Often** | **Rarely** | **Never** |
| --- | --- | --- | --- | --- | --- |
| How frequently do you use AI to prepare for your exams? |  |  |  |  |  |
| How frequently do you use AI to prepare for your homework/assignment? |  |  |  |  |  |
| How frequently do you use AI to conduct your research? |  |  |  |  |  |
| How frequently do you use AI for idea generation and brainstorming? |  |  |  |  |  |
| How frequently do you use AI for personal choices/career guidance? |  |  |  |  |  |
| How frequently do you use AI for spelling and grammar checking? |  |  |  |  |  |
| How frequently do you use AI for personality development and other skills? |  |  |  |  |  |

**Barriers**

- **Which of the following barriers restrain you from using Artificial Intelligence?**

**(you can choose more than one option)**

**Lack of knowledge and expertise**

**Lack of access/technical equipment**

**Ethical and privacy concerns**

**Lack of time due to educational burden**

**Complexity of AI**

**Limited integration in educational curricula**

**Lack of teaching centers and hands-on applications**
